# Design of the DETECT project: automated cardiac arrest detection and activation of the emergency medical chain integrated into a wristband

**DOI:** 10.1016/j.resplu.2025.100977

**Published:** 2025-05-09

**Authors:** Roos Edgar, Kambiz Ebrahimkheil, Niels T.B. Scholte, Catharina E. Jansen, Rypko J. Beukema, Marc A. Brouwer, Eelko Ronner, Aysun Cetinyurek-Yavuz, Marit van Barreveld, Marcel G.W. Dijkgraaf, Peter C. Stas, Eric Boersma, Niels van Royen, Judith L. Bonnes

**Affiliations:** a Department of Cardiology, Radboud University Medical Center, Geert Grooteplein Zuid 10, 6525 GA Nijmegen, the Netherlands; b Corsano Health, Wilhelmina Van Pruisenweg 35, 2595 AN The Hague, the Netherlands; c Department of Cardiology, Erasmus MC, University Medical Center Rotterdam, Cardiovascular Institute, Thorax Center, Dr. Molewaterplein 40, 3015 GD Rotterdam, the Netherlands; d Department of Cardiology, Reinier de Graaf Hospital, Reinier de Graafweg 5, 2625 AD Delft, the Netherlands; e Department of IQ Health, Section Biostatistics, Radboud University Medical Center, Geert Grooteplein Zuid 10, 6525 GA Nijmegen, the Netherlands; f Department of Epidemiology and Data Science, Amsterdam UMC location University of Amsterdam, Meibergdreef 9, 1105 AZ Amsterdam, the Netherlands

**Keywords:** Out-of-hospital cardiac arrest, Wearable, Automated detection, Design, Machine learning

## Abstract

**Introduction:**

While survival rates for witnessed out-of-hospital cardiac arrest have improved, assistance for unwitnessed cases often arrives too late. Automated cardiac arrest detection and alerting through wearable biosensor technology could catalyse early assistance and improve survival. Recently, an algorithm for automated detection of induced cardiac arrest was developed using wrist-derived photoplethysmography (DETECT-1a study). This paper outlines the next steps in the DETECT program, aimed at developing, validating, and preparing for the future implementation of automated cardiac arrest detection technology.

**Methods:**

External validation (sensitivity; false positives) of the PPG-algorithm developed in DETECT-1a will be performed in patients with induced shockable cardiac arrest (DETECT-1b; *n* = 50), and in cardiac arrest following withdrawal of life-sustaining treatment (DETECT-1c; n=∼50). To optimize cardiac arrest detection, DETECT-2 is set out to develop an algorithm for detection of sudden falls mimicking cardiac arrest-related collapses using accelerometry data (*n* = 20). In DETECT-3, false positive alarm rates will be studied in daily life settings, with multiple iterations to refine the algorithm: healthy volunteers/patients (*n* = ∼300) will wear the wristband for approximately two months. Finally, DETECT-4 will validate the cardiac arrest detection technology in healthy volunteers (4a; *n* = ∼50) and implantable cardioverter defibrillator-patients (4b; *n* = ∼200), assessing sensitivity and false positives. Early health technology assessment is part of the project.

**Discussion:**

The DETECT project aims to develop and validate a wristband-integrated technology for automated cardiac arrest detection and alerting in daily life settings. To support future implementation, early health technology assessment is incorporated from the outset. By providing immediate alerts, this technology has the potential to enhance rescuer response and improve cardiac arrest survival.

## Highlights

• Wearable-based cardiac arrest detection and alerting is an emerging innovation.
• The DETECT project aims to develop and validate automated cardiac arrest detection.
• Key sensor inputs include photoplethysmography and accelerometer data.
• Use of real patient data ensures accurate evaluation for real-world applicability.

## Graphical abstract

**Figure:**
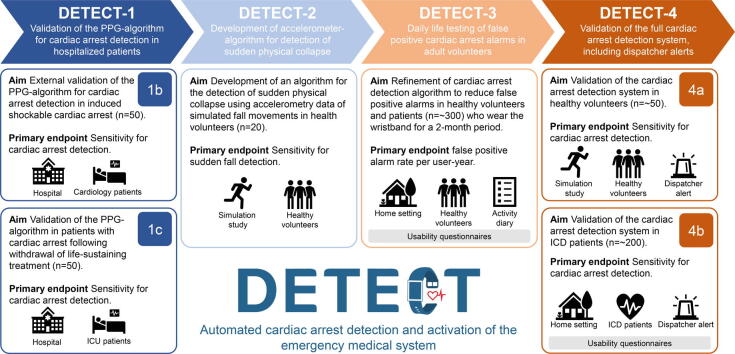

## Introduction

**Fig. 1:**
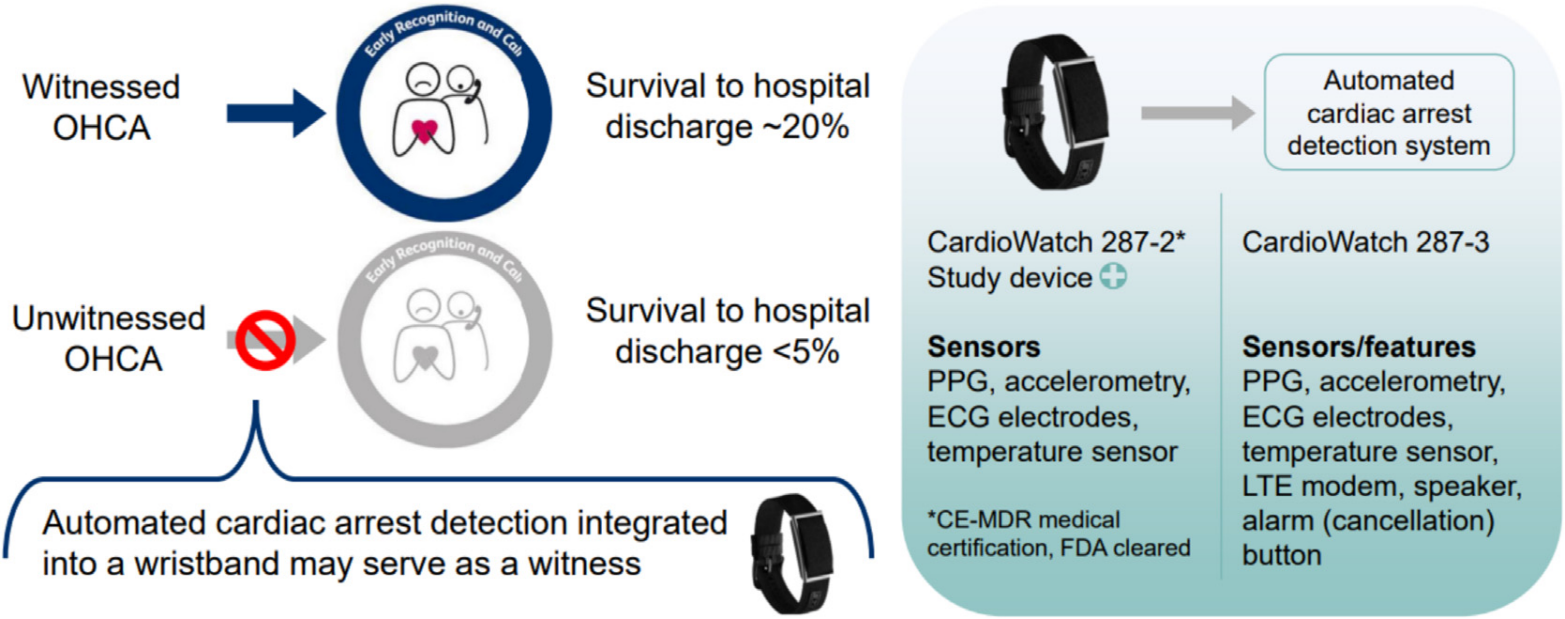
**Schematic illustration of the proposed solution for the low survival rate of unwitnessed OHCA.** The chain of survival is often not activated, resulting in poor survival chances. A wristband with automated detection of cardiac arrest may serve as a witness. The Corsano CardioWatch 287-2 wristband is being further developed to automatically detect cardiac arrest and alert the EMS.

Survival from unwitnessed out-of-hospital cardiac arrest (OHCA) remains poor (<5%), since lifesaving interventions are often initiated too late.^1^ Wearable biosensor technology automatically detecting cardiac arrest and activating the emergency medical services (EMS) could facilitate early help for victims of unwitnessed cardiac arrest (Fig. 1)^1^, ^2^, ^3^. A recent analysis estimated an increase in OHCA survival up to 2.5 times with early recognition of cardiac arrest by biosensor technologies.^4^

Several biosensors have been investigated in small studies.^5^ The most common sensor technology under investigation is photoplethysmography (PPG).^6^, ^7^, ^8^ Google recently introduced a smartwatch with ‘loss of pulse detection’ feature based on PPG and motion data, showing excellent specificity but limited sensitivity in simulation settings^9^. Other initiatives are underway.^10^, ^11^, ^12^

Among these, DETECT-1a was the first published large-scaled patient study, developing and validating an algorithm for cardiac arrest detection using wrist-derived photoplethysmography (PPG) from patients with induced cardiac arrest, achieving a 98% sensitivity.^13^ While the results were promising, it should be realized that successful future implementation warrants a highly reliable, well validated cardiac arrest detection algorithm, with excellent sensitivity and minimal false positives during daily life use to avoid unnecessary ambulance deployment. Also, practical, legal, and ethical issues surrounding implementation of this potential new technology should be addressed.

Building on the foundation established by DETECT-1a, this paper outlines the next steps of the DETECT program designed to develop, validate, and prepare for the future implementation of automated cardiac arrest detection and alarming in a daily life setting.

## Methods

### DETECT study group

**Fig. 2:**
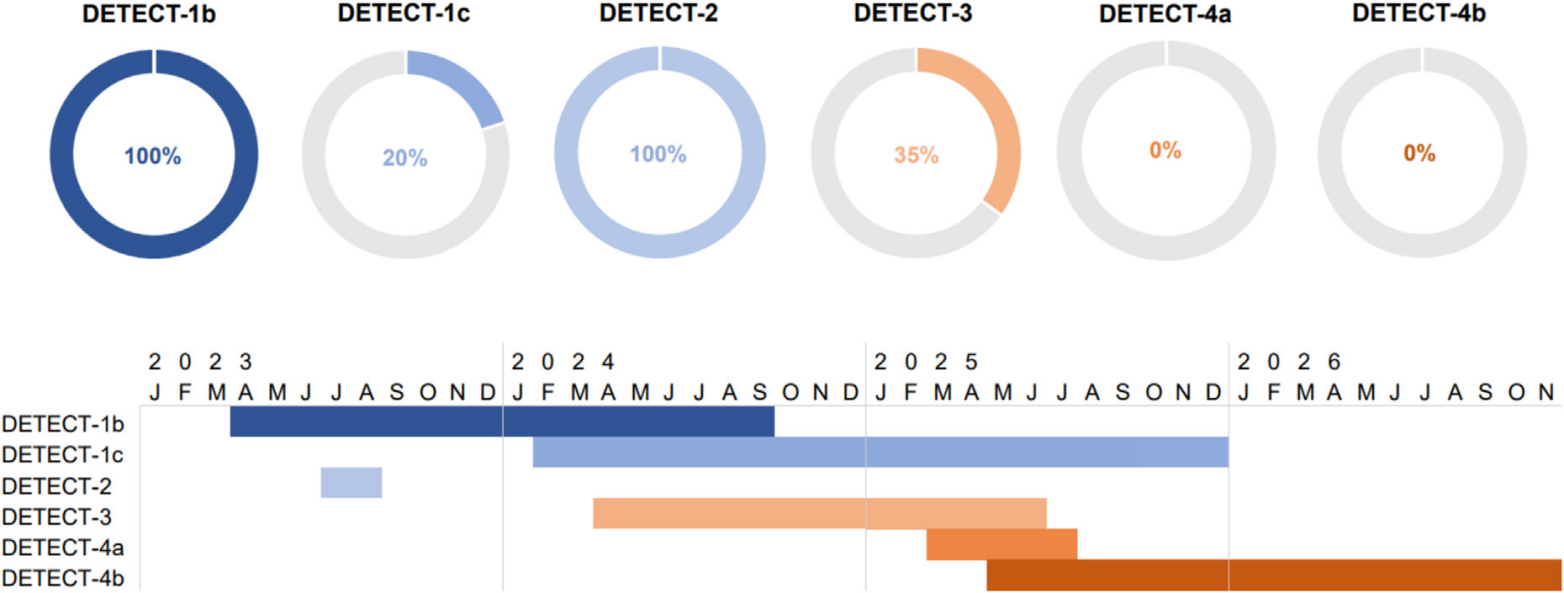
Percentage of enrolled patients, and enrolment timeline for the DETECT studies.

Research group DETECT was founded in February 2022 with the aim to develop a technological solution for automated cardiac arrest detection and alerting. The research group consists of researchers from Radboud University Medical Center (Nijmegen, the Netherlands), Erasmus MC Cardiovascular Institute, University Medical Center Rotterdam (Rotterdam, the Netherlands), Reinier de Graaf Hospital (Delft, the Netherlands), and Corsano Health (The Hague, the Netherlands). The study protocols of all DETECT studies are assessed by the Medical Research Ethics Committee Netherlands East. Informed consent is obtained from participants/relatives before inclusion. The timeline for every study and percentage of enrolled patients is depicted in Fig. 2.

### Biosensor wristband

The DETECT studies use the CardioWatch wristband (Corsano Health, The Hague, the Netherlands; Fig. 1), a remote monitoring system that incorporates PPG sensors (green, red, infrared; sample frequency 32 Hz or 128 Hz), accelerometry sensors (sampling frequency 32 Hz), electrodes for intermittent one lead ECG recordings, and a temperature sensor. Data are transmitted wirelessly to a smartphone application and then to a health cloud for storage.

In the DETECT-1 to −3 studies, we will use the CardioWatch version 287-2 to collect PPG and accelerometer data. Concurrently, a new version of the CardioWatch (287-3) will be developed that fulfills the needs for automated cardiac arrest detection and alarming. Specifications will include a speaker, manual alarm button, cancellation button, GPS, and an LTE (Long-Term Evolution) modem for direct connection to the cloud system. The 287-3 will be able to communicate the cardiac arrest alerts to the cloud directly via 5G connection. For the cardiac arrest detection algorithm, a sample frequency of 32 Hz for both the PPG and accelerometer sensor are required, yielding a battery life of approximately 4 days.

### DETECT program

The DETECT program comprises four phases (see also Graphical Abstract), during which the wristband for automated cardiac arrest detection and alerting is developed and validated. Part 1 (DETECT-1) focuses on the development and validation of the PPG-algorithm for automated cardiac arrest detection and includes studies in patients with induced shockable arrest and cardiac arrest following withdrawal of life-sustaining treatment. Part 2 (DETECT-2) examines the value of the accelerometer in detecting a sudden falls mimicking cardiac arrest-related collapse. Part 3 (DETECT-3) shifts the focus to the home environment, investigating false-positive alarms during everyday use in a broad range of users and future target groups. Part 4 (DETECT-4) validates the newly developed CardioWatch (287-3) in a simulated setting and subsequently during at-home use in ICD patients. Below, we describe the design of the studies in detail; a complete overview of the inclusion and exclusion criteria for these studies can be found in the Supplementary Material.

### PPG-algorithm development and validation in a hospital setting [DETECT-1]

#### DETECT-1b

The DETECT-1b provides external validation of the PPG-algorithm developed in DETECT-1a. The workflow of the PPG-algorithm has previously been described.^13^ DETECT-1b is a prospective multicentre study in patients with shockable cardiac arrest, conducted in four university medical centres in the Netherlands. Adult patients (*n* = 50) in whom short-lasting cardiac arrest is induced as part of routine practice during subcutaneous implantable cardioverter defibrillator (S-ICD) implantation or ventricular tachycardia (VT) ablation are eligible for inclusion. Study participants will be equipped with a PPG wristband (CardioWatch 287-2) during the procedure. As reference standards, continuous ECG and arterial blood pressure data will be collected. All events of induced ventricular fibrillation (VF) or pulseless VT, defined as a mean arterial pressure ≤ 45 mm Hg and/or pulse pressure ≤ 15 mm Hg, are regarded as cardiac arrest events. The primary endpoint is the sensitivity for cardiac arrest detection. Secondary endpoints are the positive predictive value and false positive cardiac arrest alarms.

Based on an anticipated event rate of 60% and a 98% sensitivity observed in DETECT-1a, we determined a required sample size of approximately 50 patients is required to achieve an accurate estimate of sensitivity. Using a 95% Wilson confidence interval (CI), we obtained a range of 0.833 to 0.994 with a margin of error of approximately 8%.

#### DETECT-1c

The DETECT-1c is designed to optimize and validate the PPG-algorithm in cardiac arrest following withdrawal of life-sustaining treatment, including non-shockable arrests. It is a prospective single centre study in adult patients (*n* = ∼50) in whom life-sustaining treatment is being withdrawn in the intensive care unit (ICU). After the decision to withdraw life-sustaining treatment, verbal consent is obtained from the patient (if possible) and/or the patients’ relative(s) prior to inclusion.

Patients will wear the PPG wristband (CardioWatch 287-2) during the remaining ICU stay, including the last stages of life. Routinely recorded ECG and invasive arterial blood pressure data will be collected as reference standards. The PPG-algorithm will be used to assess the sensitivity for cardiac arrest detection and the time to detection. The previously developed PPG-algorithm for cardiac arrest detection will be optimized using iterations of ten patients each, until a sensitivity of at least 95% is reached, with a maximum number of iterations of three (i.e. 30 patients). Subsequently, the algorithm will be validated using a dataset of 20 patients. This sample size calculation is based on an anticipated sensitivity of 95%. With 20 events, sensitivity can be estimated with a margin of error of approximately 11%.

### Accelerometry algorithm development [DETECT-2]

DETECT-2 is a prospective study to develop and validate an accelerometry-based algorithm for detection of sudden falls mimicking cardiac arrest related-collapses in healthy adults (*n* = 20). Each study subject will be instructed to perform a series of fall movements on a safety mat, while wearing the CardioWatch. The falls include falls forward (from a standing position or while walking), backward, sideways, and softer falls, such as sliding off a chair or against a wall. After each fall, the participant will be instructed to lay still for at least 20 s, like in a collapse resulting from cardiac arrest. The fall movements are based on previous studies investigating videotaped sudden cardiac death.^14^, ^15^, ^16^ In addition, the participants will be instructed to perform a predefined set of activities of daily living (ADL), which will be annotated as non-fall data.

Accelerometry signals will be recorded. Video recordings from the study area will be used as a reference standard. Based on the accelerometer data, a machine learning model will be developed for the detection of the sudden falls, with a 70% training set and 30% test set. Primary endpoint is the sensitivity of the accelerometry algorithm for detection of sudden falls. Secondary endpoints include false positive fall alarms and the sensitivity to detect soft falls.

The sample size will be 20 individuls, yielding a total of 400 sudden fall events (20 events per included subject), 200 soft fall events (ten per participant), and 800 simulated non-fall events (nine different types of ADLs per included subject, performed multiple times). Targeting a sensitivity of 95% for sudden fall detection, 400 sudden fall events allow estimation of sensitivity with a margin of error of approximately 2.2% (Wilson 95% confidence interval 0.946–0.967).

### Refinement of the algorithm during daily life use to reduce false positives [DETECT-3]

False positive cardiac arrest alarms experienced during daily life will be assessed in the DETECT-3 study. It is a prospective observational study in adult volunteers (n=∼300). The study population consists of the following groups: 1) Adult (healthy) volunteers in different age groups: 18–29 years, 30–39 years, 40–59 years, 60–79 years, 80 + years old; 2) Patients with a history of cardiac disease.

Participants will wear the CardioWatch 287-2 to continuously record PPG and accelerometry signals for a period of two months (at least 960 recording hours), and are asked to keep an activity diary. At the end of the study, usability will be evaluated by the Usability Metric for User Experience (UMUX), Net Promotor Score (NPS) and Autonomy and Competence in Technology Adoption (ACTA) questionnaires.^17^, ^18^, ^19^, ^20^

The collected data from the wristband will be analysed afterwards to assess the false positive cardiac arrest alarm rate using the algorithm developed in DETECT-1. After each group of 25 participants completes the study, the algorithm will be refined to reduce the false positive rate in the next iteration. This refinement includes modifications to the PPG-algorithm as well as the incorporation of accelerometer data into the cardiac arrest detection model. The false positive alarm rate (primary endpoint) is defined as the number of false positive alarms per 40 days (960 recording hours).

The PPG data from 200 participants (eight iterations of 25 participants each; ∼192,000 recording hours) will be used to train and adapt the algorithm for false positive alarms. Through software adaptations, we aim to halve the number of false positive alarms per iteration of 25 patients (covering at least 24,000 recording hours). The final set of included patients (approximately 100) will be used for algorithm validation.

### Validation of the entire CA detection system [DETECT-4]

#### DETECT-4a

DETECT-4a is designed to test the performance of the developed cardiac arrest detection model − which now integrates both PPG and accelerometer data − in simulated cardiac arrest scenarios, providing final optimization and validation. It is a simulation study in healthy adult volunteers (n=∼50) in whom we simulate cardiac arrest by two steps, while wearing the CardioWatch (model 287-2/287-3). First, a circulatory arrest will be simulated by inflating a blood pressure cuff on the same arm as where the wristband is applied. Second, sudden falls mimicking cardiac arrest related-collapses will be performed by the participant immediately after circulatory arrest induction (at least eight simulated collapses per person). The simulated collapses will either be sudden or gently, from standing, sitting, and lying positions.

The collected PPG and accelerometry data will be used for the final optimization of the previously developed cardiac arrest detection model using iterations of ten participants each (at least 80 simulated cardiac arrest events per iteration), until a sensitivity of at least 95% is reached, with a maximum of four iterations (i.e. 40 participants; at least 320 simulated events). Subsequently, prospective testing will be performed in a final subset of ten participants with at least 80 simulated arrests. Considering the non-normal distribution of the sensitivity estimate, the Wilson 95% CI is approximately 0.93–0.98 with a margin of error of ± 5%.

#### DETECT-4b

The DETECT-4b study is a prospective multicentre observational cohort study in which the complete cardiac arrest detection system will be validated in ICD-patients (*n* = ∼200) during daily life use. The cardiac arrest detection system consists of the cardiac arrest detection algorithm integrated into the CardioWatch 287-3 wristband and includes test alerting to the dispatch centre.

Adult patients with an ICD for secondary prevention and patients with an ICD for primary prevention who have received at least one time appropriate ICD therapy are eligible for inclusion. Participants will wear the CardioWatch 287-3 for one to two years. The primary endpoint is the sensitivity for the detection of a first episode of cardiac arrest. Secondary endpoints are the positive predictive value, false positive alarms per user year, day-level specificity, and sensitivity for the detection of all cardiac arrest events. A cardiac arrest event is defined as appropriate ICD therapy in combination with (short-lasting) loss of consciousness, transient loss of consciousness with a ventricular arrhythmia in the monitoring zone or discharge diagnosis of presumed ventricular arrhythmias below the ICD monitor/detection zone, or true death. Intracardiac electrogram recordings in combination with clinical reports will be used as reference standard. Usability will be studied using the UMUX, NPS and ACTA questionnaires.^17^, ^18^, ^19^, ^20^

The presumed incidence of cardiac arrest events (as defined above) is expected to be 8% per year.^21^, ^22^ Given an expected median follow-up duration of 1.5 years and assuming a true sensitivity of 95%, a sample of approximately 200 patients is needed to achieve this sensitivity with a 95% Wilson confidence interval ranging from 74% to 99%, corresponding to a margin of error of ± 12%. Since the number of sampled non-events is substantially larger, specificity can likewise be confirmed with sufficient power. The DETECT-4b is registered at ClinicalTrials.gov (NCT06692374).

### Early HTA

Early HTA will be performed to assess the potential impact of the technology to be developed and prepare future implementation. Early, model-based economic evaluation given expected costs and health effects will be performed to explore the probability of the CardioWatch 287-3 being cost-effective for (i) distinct target populations in comparison with target group specific alternative treatments, and at (ii) different levels of device unit costs and (iii) different levels of willingness to pay per quality adjusted life year. Both, healthcare and societal perspectives will be addressed to gain sufficient insights to adequately design a full economic evaluation alongside future effectiveness research. Patients and the public are consulted to evaluate patient satisfaction and impact on quality of life. Dispatchers and the Dutch lay rescuer network are involved to assess key organizational elements and prepare future implementation. Test alerts are being conducted during DETECT-4.

## Discussion

The DETECT project is designed to develop and validate a comprehensive wearable-based system for automated cardiac arrest detection and alerting of the EMS in a daily life setting. The studies utilize both simulated and real patient data, progressing from controlled study settings to real-world conditions. An early HTA is conducted to prepare for future implementation. Rapid localization of the cardiac arrest victim and connection with lay and professional rescuers are central features of the wearable as timely assistance is essential to enable this technology to provide survival benefits.

**Fig. 3:**
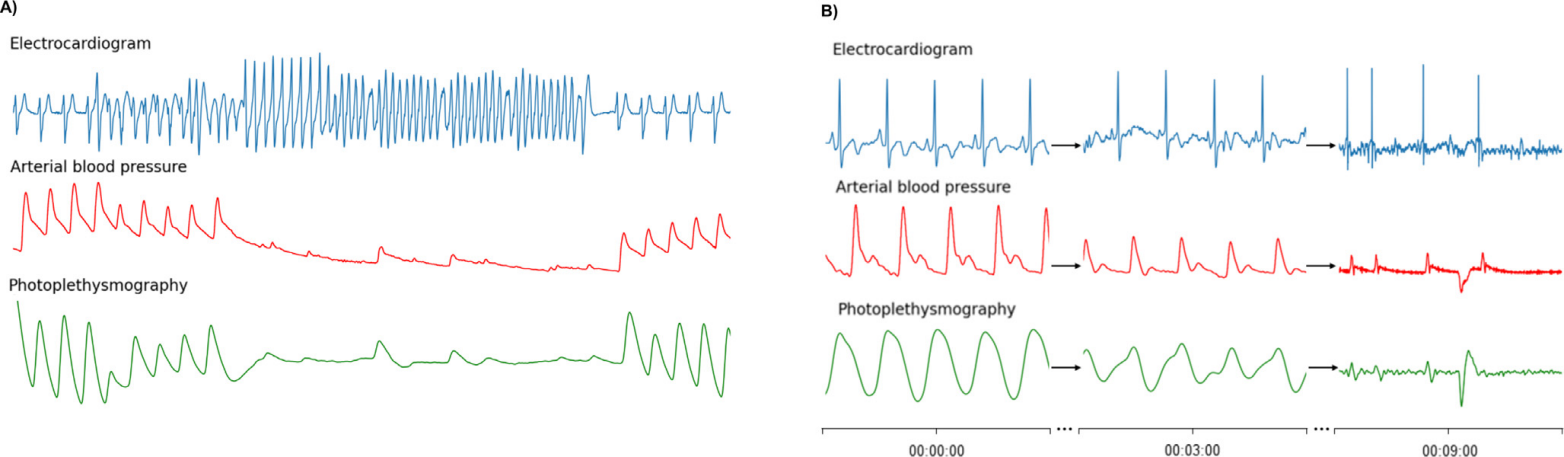
**Data from a shockable and non-shockablecardiac arrestevent.** A) Induced short-lasting shockable arrest in a patient undergoing VT-ablation. When a VT is induced on the ECG, the arterial blood pressure and PPG signal flatten. B) Cardiac arrest based on asystole in a patient admitted to the ICU in whom life sustaining treatment has been withdrawn. The initial recording (on the left) shows sinus rhythm with a blood pressure of 110/70 mmHg and a normal pulsatile PPG signal. The panel in the middle, after three minutes, shows blood pressure of 60/40 mmHg and a decreased amplitude of the PPG signal. The panel on the right, after nine minutes, shows the final pulsations with an arterial blood pressure of 25/20 mmHg after which asystole occurs. VT = ventricular tachycardia, ECG = electrocardiogram, ICU = intensive care unit, PPG = photoplethysmography.

In the DETECT program, we aim for high sensitivity as the device is intended for patients in whom missing a cardiac arrest is not acceptable. DETECT-1c will provide the first-ever assessment of wearable cardiac arrest detection technology in cardiac arrest following withdrawal of life-sustaining treatment, including both shockable and non-shockable rhythms. As PPG measures blood flow rather than electrical activity as the ECG does, both arrest types will result in a flattering of the PPG signal at the wrist and are expected to be well detected. Related to the underlying causes, asystole and PEA may be preceded by hemodynamic instability whereas VT/VF typically occurs sudden (Fig. 3); data of the DETECT-1c study including non-shockable cardiac arrests will be used to fine-tune the timing of the cardiac arrest alarm in these cases.

Practical implementation of automated cardiac arrest detection demands very few false positive alarms to prevent unnecessary ambulance dispatches. False positive cardiac arrest alarms will be addressed in the DETECT-3 study. The study uses multiple iterations to refine the algorithm and provide insight into the impact of various adaptations. One planned software change involves prolonging the detection interval, which was limited to five seconds in the DETECT-1a study since the induced cardiac arrest episodes were short-lasting. In contrast, the Google Pixel Watch with integrated loss of pulse detection takes 77 s before the emergency call.^9^ A detection interval longer than five seconds is clinically reasonable and expected to reduce false alarms. Incorporating accelerometer data is also expected to play a key role, as cardiac arrest typically results in a physical collapse and is incompatible with continued user movement. In terms of hardware, integrating a speaker for audio alarms and including a cancel button on the bracelet allows users to manually cancel false alarms, further reducing the false positive rate.

In the DETECT-4 studies, the complete cardiac arrest detection system will be tested. This includes evaluating the connection between the bracelet and the dispatch centre. When a cardiac arrest is detected, the bracelet will emit an acoustic alarm and alerts the dispatch centre in case of a non-cancelled alert. Despite all these efforts, the false alarm rate will not be reduced to zero. False positive alerts are also observed in systems like crash detection in cars and crash or fall detection in smartwatches and smartphones,^23^ but must be minimized to avoid unnecessary ambulance dispatches. While false alarms are a concern, automated cardiac arrest detection can also contribute to more accurate alerts. It is known that EMS dispatchers can sometimes misinterpret the presence or absence of cardiac arrest during phone assessments.^24^, ^25^ Therefore, automated cardiac arrest detection not only has the potential to serve as first witness but can also confirm or correct a human-reported cardiac arrest.

The DETECT program distinguishes itself from other initiatives by prioritizing patient data over simulated data and using sufficiently large sample sizes to ensure a robust scientific foundation for the technology.^10^, ^11^, ^12^ The goal is to achieve high sensitivity (>90–95%) to minimize the risk of missing a cardiac arrest diagnosis, while keeping false positives low to support feasible implementation. The design of the DETECT program encompasses all necessary steps for the development, validation, and testing of cardiac arrest detection technology. The successive studies will yield comprehensive insights into the factors influencing its accuracy guiding further development. Once available, post-implementation and effectiveness studies need to be conducted to provide evidence of the value of wearable-based cardiac arrest technologies in improving patient outcomes.

## CRediT authorship contribution statement

**Roos Edgar:** Writing – review & editing, Writing – original draft, Visualization, Validation, Project administration, Methodology, Investigation, Conceptualization. **Kambiz Ebrahimkheil:** Writing – review & editing, Visualization, Validation, Software, Investigation, Conceptualization. **Niels T.B. Scholte:** Writing – review & editing, Methodology, Investigation, Conceptualization. **Catharina E. Jansen:** Writing – review & editing, Investigation, Conceptualization. **Rypko J. Beukema:** Writing – review & editing, Supervision, Funding acquisition, Conceptualization. **Marc A. Brouwer:** Writing – review & editing, Supervision, Funding acquisition, Conceptualization. **Eelko Ronner:** Writing – review & editing, Supervision, Investigation, Funding acquisition, Conceptualization. **Aysun Cetinyurek-Yavuz:** Writing – review & editing, Methodology, Investigation, Conceptualization. **Marit van Barreveld:** Writing – review & editing, Methodology, Investigation, Conceptualization. **Marcel G.W. Dijkgraaf:** Writing – review & editing, Methodology, Investigation, Conceptualization. **Peter C. Stas:** Writing – review & editing, Funding acquisition, Supervision, Software, Validation, Resources, Methodology, Investigation, Conceptualization. **Eric Boersma:** Writing – review & editing, Supervision, Methodology, Investigation, Funding acquisition, Conceptualization. **Niels van Royen:** Writing – review & editing, Validation, Supervision, Resources, Project administration, Methodology, Investigation, Funding acquisition, Conceptualization. **Judith L. Bonnes:** Writing – review & editing, Writing – original draft, Validation, Supervision, Resources, Project administration, Methodology, Investigation, Funding acquisition, Conceptualization.

## Funding

This research project is financed by public–private partnerships allowance (grant number 2021B006; grant number R0007420; grant number R0007706) made available by Top Sector Life Science & Health to Hartstichting (Dutch Heart Foundation) and Radboudumc to stimulate public–private partnerships.

## Declaration of competing interest

The authors declare the following financial interests/personal relationships which may be considered as potential competing interests: Niels van Royen reports financial support was provided by Netherlands Heart Foundation. Niels van Royen reports financial support was provided by Radboud University Medical Center. Eelko Ronner reports a relationship with Corsano Health that includes: consulting or advisory. Niels van Royen reports a relationship with Biotronik that includes: funding grants. Niels van Royen reports a relationship with Abbott that includes: funding grants. Niels van Royen reports a relationship with Medtronic that includes: funding grants. Niels van Royen reports a relationship with Philips that includes: funding grants. Niels van Royen reports a relationship with Bayer that includes: speaking and lecture fees. Niels van Royen reports a relationship with RainMed that includes: speaking and lecture fees. Niels van Royen reports a relationship with Microport that includes: speaking and lecture fees. Roos Edgar is a member of the Young Investigators Editorial Board of Resuscitation Plus. If there are other authors, they declare that they have no known competing financial interests or personal relationships that could have appeared to influence the work reported in this paper.
