# Supplementary material for: Design of the DETECT project: automated cardiac arrest detection and activation of the emergency medical chain integrated into a wristband
Source: Resusc Plus. 2025 May 9;24:100977. doi: 10.1016/j.resplu.2025.100977 (PMC12148417; doi:10.1016/j.resplu.2025.100977)
Supplement: Supplementary Data 1 [file mmc1.docx]

**Supplement – Inclusion and exclusion criteria for the DETECT studies**

**DETECT-1b**

Inclusion criteria

- Age 18 years or older
- A subject must meet one of the following criteria
  - Planned to undergo ventricular fibrillation induction during defibrillation testing after ICD implantation
  - Undergoing ventricular tachycardia ablation

Exclusion criteria

- Known bilateral significant subclavian artery stenosis.
- Medical issues interfering with the wearing of the wristband (e.g. skin disorders).

**DETECT-1c**

Inclusion criteria

- Age 18 years or older
- Planned withdrawal of life sustaining therapy at the intensive care unit
- Having an arterial line inserted for invasive blood pressure monitoring

Exclusion criteria

- The absence of an arterial line for blood pressure monitoring.
- Known bilateral significant subclavian artery stenosis.
- Medical issues interfering with the wearing of the wristband (e.g. skin disorders).

**DETECT-2**

Inclusion criteria

- Age 18 years or older

Exclusion criteria are

- (Physically) unable or unwilling to perform the (fall-)motions.
- Known significant bilateral subclavian arterial stenosis.
- Medical issues that interfere with wearing of the wristband (e.g. skin disorders).

**DETECT-3**

Inclusion criteria, belonging to one of the groups below:

- (Healthy) volunteers in the age group between 18 and 80+ years old.
- Intended target groups consisting of outpatients at increased risk of sudden cardiac death, including post-myocardial infarction patients, patients with heart failure with reduced ejection fraction; patients with previous ventricular arrhythmias including ICD-patients, patients with a clinically-diagnosed cardiomyopathy, and patients with a positive family history for sudden cardiac death.

Exclusion criteria

- Not having a (compatible) smartphone.
- Insufficient skills to operate the device/app.
- Known hemodynamically relevant bilateral subclavian artery stenosis.
- Medical issues interfering with wearing of the wristband (e.g. skin disorders).

**DETECT-4a**

Inclusion criteria

- Age 18 years or older.

Exclusion criteria

- (Physically) unable or unwilling to perform the (fall-)motions.
- Medical issues that interfere with wearing the inflated blood pressure cuff for a prolonged period, such as pre-existing pain, neurological symptoms or history of injury within the past three months in either upper extremity.
- Known bilateral significant subclavian artery stenosis.
- Medical issues that interfere with wearing of the wristband (e.g. skin disorders)

**DETECT-4b**

Inclusion criteria

- Age 18 years or older.
- Patients with an;
  - ICD for secondary prevention.
  - ICD for primary prevention who received at least one appropriate ICD shock in the past 5 years.

Exclusion criteria

- Not having a smartphone.
- Insufficient skills to operate the device/app.
- Known hemodynamically relevant bilateral subclavian artery stenosis.
- Medical issues that interfere with wearing of the wristband (e.g. skin disorders).
